# Developmental phenotype in Phelan-McDermid (22q13.3 deletion) syndrome: a systematic and prospective study in 34 children

**DOI:** 10.1186/s11689-016-9150-0

**Published:** 2016-04-26

**Authors:** Renée J. Zwanenburg, Selma A.J. Ruiter, Edwin R. van den Heuvel, Boudien C.T. Flapper, Conny M.A. Van Ravenswaaij-Arts

**Affiliations:** University of Groningen, University Medical Center Groningen, Department of Genetics, Hanzeplein 1, 9713 GZ Groningen, The Netherlands; De Kinderacademie Groningen, Centre of Expertise for Child Development Care and Research, Herestraat 106, 9711 GH Groningen, The Netherlands; Eindhoven University of Technology, Department of Mathematics and Computer Science, Den Dolech 2, 5612 AZ Eindhoven, The Netherlands; University of Groningen, University Medical Center Groningen, Department of Pediatrics, Hanzeplein 1, 9713 GZ Groningen, The Netherlands

**Keywords:** Phelan-McDermid syndrome, 22q13 deletion syndrome, *SHANK3*, Intellectual disability, Autism, Neurodevelopmental disorders, Developmental phenotype

## Abstract

**Background:**

Phelan-McDermid syndrome (PMS) or 22q13.3 deletion syndrome is characterized by global developmental delay, cognitive deficits, and behaviour in the autism spectrum. Knowledge about developmental and behavioural characteristics of this rare chromosomal disorder is still limited despite a rapid growing number of diagnoses. Our aim was to study a new and relatively large cohort to further characterize the developmental phenotype of children with PMS.

**Methods:**

We performed a descriptive study of children with a 22q13.3 deletion including *SHANK3*, aged 8 to 178 months, who were systematically (*n* = 34) and longitudinally (*n* = 29) assessed with standardized instruments: Bayley Scales of Infant and Toddler Development, third edition; Wechsler Preschool and Primary Scale of Intelligence, third edition; and Vineland Screener for Social and Adaptive Behavior.

**Results:**

Maximal developmental functioning ranged from 34 to 52 months depending on the developmental domain. In general, children performed poorest in the domain of language and best on the domain of motor (young children) or cognitive development (older children). At the individual level, 25 % scored better for receptive and 18 % for expressive language, whereas 22 % scored better for fine and 33 % for gross motor function. Developmental quotients were higher in younger children and decreased with age for all developmental domains, with 38 % of the children showing no improvement of cognitive developmental functioning. Almost all children (33/34) had significant deficits in adaptive behaviour. Children with very small deletions, covering only the *SHANK3*, *ACR*, and *RABL2B* genes, had a more favourable developmental phenotype.

**Conclusions:**

Cognitive, motor, and especially language development were significantly impaired in all children with PMS but also highly variable and unpredictable. In addition, deficits in adaptive behaviour further hampered their cognitive development. Therefore, cognitive and behavioural characteristics should be evaluated and followed in each child with PMS to adapt supportive and therapeutic strategies to individual needs. Further research evaluating the relationship between deletion characteristics and the developmental phenotype is warranted to improve counselling of parents.

**Electronic supplementary material:**

The online version of this article (doi:10.1186/s11689-016-9150-0) contains supplementary material, which is available to authorized users.

## Background

Phelan-McDermid syndrome (PMS; MIM# 606232) is one of the more common microdeletion syndromes (deletion 22q13.3) with hundreds of individual diagnoses made worldwide (Phelan-McDermid Syndrome Foundation, Venice, FL) since it was first reported in 1985 [1]. To our knowledge, there are at least 60 children and 30 adults currently diagnosed in the Netherlands based on data supplied by the eight genetic centres in the Netherlands. These numbers are likely to be underestimates due to the non-specific physical features of PMS, but the number of diagnosed individuals has rapidly increased since the introduction of non-targeted microarray techniques in diagnostics. Children with PMS have a global developmental delay with cognitive deficits, behavioural problems in the autism spectrum and mild dysmorphic features. Some children also have congenital kidney anomalies and neurological problems such as abnormal movement patterns, seizures, reduced sensitivity to pain, and an inability to regulate sweating [2]. The syndrome is still under-diagnosed in adults with intellectual disability, who may present with deterioration in cognitive functioning and atypical bipolar disorders with loss of acquired skills [3–5]. The extensive cognitive and behavioural and physical problems associated with this disorder are a social and emotional challenge for patients and their families.

The cause of PMS is a microdeletion of the distal q-arm of chromosome 22, within band q13.3 [6]. The most common cause is a terminal deletion that varies in size from 100 kb to over 9 Mb. It can be the result of a pure terminal deletion, secondary to an unbalanced translocation or due to a ring chromosome 22 [7]. Currently, the hypothesis is that deletion of the *SHANK3* gene, located on 22q13.3, is the major contributor to the neurological features of this syndrome [8]. The SHANK3 protein is an important scaffold in the post-synapses of neurons in areas of the brain that are important for learning and cognition as well as for communication [9–11]. Individuals with an intragenic deletion or a mutation of *SHANK3* share the features of intellectual disability and autistic behaviour [12–14], making 22q13.3 deletion syndrome a widely studied model for autism. However, other genes probably contribute to the phenotype of PMS because patients with interstitial deletions not involving SHANK3 share similar clinical features [15, 16]. In terminal deletions with a size above 6.7 Mb, the *PARVB* gene and the nearby upstream-located *SULT4A1* gene have been suggested to contribute to phenotype severity [16]. Genotype-phenotype studies in larger populations would contribute to a better resolved definition for PMS. For this study, we define PMS as those patients with a terminal 22q13.3 deletion including *SHANK3*.

Unfortunately, detailed information about developmental characteristics in PMS is still limited and incomplete. Kolevzon and colleagues provided a useful overview of practice parameters for medical care of individuals with PMS, but cognitive and behavioural assessments did not come within the scope of their paper [17]. To date, only three prospective or systematic studies have been published in which development and autistic behaviour are tested in a standardized manner and in a larger sample instead of individual case reports [6, 18, 19]. These studies report a highly variable level of cognitive functioning with the majority of patients presenting with moderate to severe intellectual disability [6, 19]. The most affected developmental domain is language, although receptive language is usually reported to be stronger than expressive language. The least-affected domain is motor functioning, with gross motor function being stronger than fine motor function [18, 19]. However, these few cross-sectional studies did not perform follow-up studies. Nor did they take into account the effect of age at testing and individual deletion characteristics. The age at testing is important to consider because developmental functioning of children with intellectual disability changes with age, with the difference compared to typical peers increasing over time [20]. These studies also did not report on the influence of autistic features like a lack of communication, socialization, and adaptive behaviour, which can prevent children from acquiring new skills and therefore hamper their developmental potential [21]. As a result, the reported level of developmental functioning can be an under- or overestimation depending on the mean age at testing and the presence of autistic behaviour in the study sample. More detailed information on developmental characteristics in children with PMS is clearly necessary to assist paediatricians, psychiatrists, and other health care professionals involved in the care of these children. This information would contribute to improved counselling of parents, identification of specific problems, and better organization of adequate and individualized support for individuals with PMS.

The aim of this study was to systematically and longitudinally assess development in a new and relatively large cohort of children with PMS. This descriptive study not only focuses on different domains of development, e.g. cognitive, language, and motor development, but also evaluates the age at testing, deficits in adaptive behaviour and deletion size. Moreover, we present the first follow-up data in 29 individuals with PMS. The data presented here contribute to and expand upon the knowledge of the developmental phenotype of children with PMS.

## Methods

### Study participants

Participants for this descriptive study were recruited among children with 22q13.3 deletion syndrome diagnosed at the Clinical Genetics department of the University Medical Centre Groningen or referred from other university medical centres in the Netherlands. All diagnoses of a 22q13.3 deletion were made by molecular techniques. Thirty children had already participated in independent studies performed by the same project group (protocol ID 2009/251 and 2012/329), and developmental data from these studies are used in this study. Four children visited our expert clinic for Rare Chromosome Disorders in Groningen and received developmental tests during regular clinical care. Two trained psychologists experienced in working with children with special needs performed developmental assessments of the children at their home, day-care or, for four patients, at the outpatient clinic in Groningen. Individual data were collected from patient histories obtained from parents and from clinical data from medical files. Parents provided written informed consent. The Medical Ethical Review Board of the University Medical Center Groningen (UMCG, the Netherlands) approved this study.

### Assessment of cognitive, language, and motor development

To assess developmental functioning, the Bayley Scales of Infant and Toddler Development, third edition, adapted and validated for the Dutch population (Bayley-III-NL), were used [22]. This instrument assesses developmental functioning in the domains of cognition, receptive and expressive communication, and fine and gross motor development. It is applied to infants and young children aged 1 month to 42 months but has also been validated in older children suspected to have developmental functioning within this calendar age range [23]. Raw scores of each Bayley-III-NL developmental domain were converted into a mental age, or developmental age-equivalent (DAE), in months. In typical children, this DAE approximately equals their chronological age (CA). To investigate the level of developmental functioning of our participants in relation to their expected level of developmental functioning based on their calendar age, we calculated the DAE/CA ratio at time of assessment. This ratio was multiplied by 100 to result in a developmental quotient (DQ) for each domain [24–26]. Typical children have a DAE/CA ratio of approximately 1 and a DQ of 100 (range 85–115). One child in our cohort (individual 31) was initially assessed with the Bayley-III-NL but obtained maximal scores. In order to estimate the level of cognitive functioning, she was additionally assessed with the Wechsler Preschool and Primary Scale of Intelligence, third edition, Dutch version (WPPSI-III-NL) [27]. The WPPSI-III-NL is an intelligence test designed for children aged 30 to 95 months and assesses general cognitive functioning (full scale IQ (FSIQ)), verbal functioning (verbal intelligence quotient (VIQ)), and performal functioning (performal intelligence quotient (PIQ)). Raw scores of the general cognitive functioning were converted into a DAE and used to calculate the DQ as described above. Results of other WPPSI-III-NL domains cannot be compared to Bayley-III-NL domains and will not be described.

### Developmental follow-up

Data from children, who were assessed twice with an interval of at least 5 months, were used to investigate the natural course of development: developmental growth (DG). DG is the change in developmental age (DAE) between the first and second assessment. To enable comparison of the differences between the two measurements (DG) among the different children, DG is calculated and expressed as change in developmental age in months over a period of 6 months. In clinical practice, an interval of at least 1 year is usually used as the evaluation period. However, an interval of 6 months is more suitable to future therapeutic interventions like our clinical trial with intranasal insulin [28]. In addition, a test-retest effect is not expected over 6 months in these cognitively impaired children. DG is calculated as the ratio of change in DAE and change in CA (per month), multiplied by six: ((DAE_2_ − DAE_1_)/(CA_2_ − CA_1_)) × 6. A DG ≤ 0 represents no improvement, DG = 6 normal development and DG > 6 above average development.

### Assessment of adaptive behaviour

To estimate the level of functioning in adaptive behaviour, we used the Vineland Screener 0–6 years [29]. This test is based on the American short version of the Vineland Adaptive Behavior Scales (VABS) [30]. The Vineland screener 0–6 years has been developed and validated in the Netherlands for children aged 0 to 6 years and also for older children with a developmental age corresponding to the calendar age range of the Vineland Screener [29, 31–33]. It uses a limited number of items from the four domains of adaptive behaviour portrayed in the original Expanded Version of the VABS. For our study, we used the domains of communicative, social, and daily skills. To express the level of developmental functioning of adaptive behaviour, raw scores of each VABS developmental domain were converted into a DAE in months and a DQ was calculated as described above.

### Effect of deletion size and gender

Children were divided into three groups of different deletion size to investigate the hypothesis that children with very small deletions function at a higher level. Group 1 consisted of children with a deletion smaller than 250 kb (including only three common OMIM genes: *SHANK3*, *ACR*, and *RABL2B*). This group also included individual 31, who was assessed with the WPPSI-III-NL. The remaining children were divided into groups 2 and 3, separated by a boundary at 6.7 Mb, which is downstream of *PARVB*.

We also explored the relation between gender and cognitive developmental functioning, by dividing children based on gender.

### Data analysis

The first successful measurement of each domain was used to illustrate individual DAEs and DQs. DAEs and DQs were plotted for each Bayley domain. To compare mean DAEs and DQs at different ages, children were divided into four age groups: 0–35.9, 36–71.9, 72–107.9, and ≥108 months. Mean standard deviations and ranges were calculated for deletion size, DAE, DQ, and DG. GraphPad Prism 5 software was used for these figures and calculations (GraphPad Software, Inc., La Jolla, CA).

Outcome cognitive DAE was modelled as a function of chronological age using non-linear mixed models. It was assumed that the ratio of DAE and chronological age follows a one-parameter Michaelis-Menten curve, i.e. DAE/CA = 1 − CA/(*θ* + CA). The parameter *θ* was considered subject-specific to address the correlation among repeated results of individual children. Theta represents the chronological age at which a child drops to a DQ of 50. A likelihood ratio test was applied to investigate whether *θ* depends on deletion group and whether there is a difference between the deletion groups. A *p* value <0.05 is considered significant. The statistical analysis was done with SAS software, version 9.4 (SAS Institute Inc., Cary, NC).

## Results

### Characteristics of children

Characteristics of children are summarized in Table 1. Developmental data were collected for 34 Dutch children, 9 males and 25 females, with ages at first assessment of 8.1 to 178.1 months (median 55.7, mean 68.4 months). Twenty-nine children received a second assessment after 5.2 to 16.1 months (median 10.0, mean 9.5 months).

**Table 1:** Characteristics of children with a 22q13.3 deletion

Patient	Age 1st test (mo)	Age 2nd test (mo)	Gender	Deletion type	Deletion size (Mb)	Walking unassisted (mo)	Medication at 1st assessment
1	8	13	M	Terminal	6.5	25 (crawling)	None
2	10	16	F	Terminal	2.1	12	None
3	11	22	F	Terminal	1.9	19	None
4	16	27	F	Terminal	7.7	36	Salbutamol (as needed)
5	17	23	F	Terminal + dup 13q (2.3 Mb)	7.3	39 (walking assisted)	None
6	17	n.a.	F	Terminal	9.2	12 (rolling over)	None
7	35	n.a.	M	Terminal^a^	4.4	21	Not reported
8	37	46	F	Terminal	3.2	30	Valproic acid (for absence like periods)
9	37	45	M	Terminal	2.1	17	None
10	39	n.a.	F	Terminal	182 kb	16	Not reported
11	42	48	F	Terminal	587 kb	24	Macrogol and omeprazole
12	42	53	F	Terminal	6.2	27	None
13	43	49	F	Terminal^b^	6.6	20	None
14	44	55	F	Terminal	7.4	76	None
15	46	62	F	Terminal	6.2	25	None
16	46	57	F	Terminal + del 16p (761 kb)	3.0	42	Beclometason dipropionate, salbutamole, ipratropium bromide
17	47	57	M	Terminal	182 kb	18	Risperidone and clonidine
18	64	n.a.	F	Terminal	183 kb	16	Macrogol
19	65	75	F	Ring 22	2.3	23	None
20	72	82	F	Terminal	1.6	17	None
21	83	96	M	Ring 22	3.1	28	None
22	89	99	F	Ring 22	3.4	31	None
23	92	101	M	Ring 22	2.7	24	None
24	92	n.a.	F	Terminal^c^	n.a.	n.a.	Not reported
25	95	109	M	Terminal	6.1	43	Melatonin
26	95	105	F	Terminal	6.4	96	Omeprazole, alginic acid, domperidone, trimethoprim, melatonin
27	109	119	F	Terminal	377 kb	16	Melatonin
28	112	118	F	Terminal + dup 12q (5.1 Mb)	2.0	22	None
29	113	123	M	Terminal	7.8	48	None
30	118	129	F	Ring 22	3.4	32	None
31	119	126	F	Terminal	224 kb	15	None
32	142	150	M	Terminal	5.0	24 (walking assisted)	Alimemazine and melatonin
33	147	157	F	Terminal	3.5	32	None
34	178	189	F	Terminal	5.7	19	Lamotrigine (for fever-induced convulsions)

*mo* months, *n.a.* not available, *M* male, *F* female, *dup* duplication, *del* deletion

^a^Mosaic deletion (in 25 % of peripheral blood cells)

^b^Mosaic of 6.6 and 6.2 Mb deletion (in 80–85 % and 15–20 % of cells, respectively)

^c^Diagnosis with FISH only

In 33 children, 22q13.3 deletions were diagnosed and characterized by high-resolution array. In one child, the diagnosis of a terminal 22q13.3 deletion was made by fluorescent in situ hybridization (46,XX,del(22)(q13.1).ish del(22)(SHANK3-,GS-99 K24-)) and the exact deletion size is unknown. Deletion sizes ranged from 182 kb to 9.2 Mb (UCSC Genome Browser, hg19 reference genome, reference to last probe position 51304566).

**Fig. 1:**
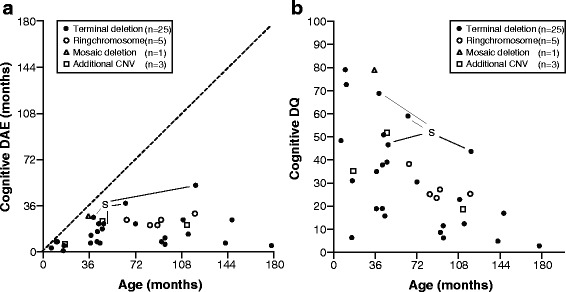
Developmental age-equivalents (DAE) (**a**) and developmental quotients (DQs) (**b**) of children with 22q13.3 deletions. Data based on Bayley-III-NL (*n* = 33) and WPPSI-III-NL (*n* = 1) results for cognition. The *dashed line* represents the normal relation between DAE and age in typically developing children. *Terminal deletion* terminal deletion not caused by ring chromosome formation. Mosaic deletion (individual 7), *additional CNV* additional copy number variation (individuals 5,16, 28), *S* small deletion including only *SHANK3*, *ACR*, and *RABL2B* (individuals 10, 17, 18, 31)

Four children had a small deletion of 182 to 224 kb that includes only three OMIM genes: *SHANK3*, *ACR*, and *RABL2B.* Twenty-three children had a medium-sized deletion ranging from 377 kb to 6.6 Mb, and five children had a larger deletion ranging from 7.3 to 9.2 Mb extending beyond the *PARVB* gene*.* Three children had an additional copy number variation (CNV) of another chromosome (individuals 5, 16, and 28), five children had a deletion caused by a ring chromosome 22 (individuals 19, 21, 22, 23, and 30) and one child had a mosaic terminal deletion (individual 7). Because the effect of a mosaic deletion is unknown, results of the latter individual were only summarized in Table 1 and depicted in Fig. 1 and Additional file 1: Fig. S1 but excluded from further analyses and group descriptions.

### Cognitive, language, and motor development

Thirty-two children were assessed with the Bayley-III-NL and one child with the WPPSI-III-NL (individual 31). Chronological ages were corrected for prematurity at first and second assessments (individual 1: 6.4 and 11.5 months, individual 6: 15.6 months).

There is a high variability of cognitive DAEs and DQs within each age group (Fig. 1). Children with a very small deletion (<250 kb) or a ring chromosome 22 (deletion size 2.3–3.4 Mb) had higher DAEs and DQs compared to other children within the same age group. Children with an additional CNV did not perform worse than other affected children within their age group. Development of cognition appeared to increasingly deviate from normal development (Fig. 1a, dashed line) in older children, as also indicated by a decreasing DQ with age (Fig. 1b). In fact, mean DAEs did increase between age groups 1 (0–35.9 months) and 2 (36–71.9 months) but did not further increase in age groups 3 (72–107.9 months) and 4 (≥108 months) (Table 2). The highest level of cognitive functioning for children assessed with the Bayley-III in this cohort was 38 months. One child (individual 31) was initially assessed with the Bayley-III-NL. Her results revealed maximum DAEs on all domains so her developmental functioning was beyond the developmental age range of the Bayley-III-NL. Therefore, she was additionally assessed with the WPPSI-III-NL. Converting her WPPSI-III-NL raw scores to a DAE showed a cognitive DAE of 52 months with a corresponding DQ of 44.

**Table 2:** Bayley-III-NL developmental age-equivalents (DAEs) and developmental quotients (DQs) for each age group

Developmental domain		Age group 1 0–35.9 months	Age group 2 36–71.9 months	Age group 3 72–107.9 months	Age group 4 ≥108 months	All ages
Cognition^a^	Number	6	12	7	7	32
	Mean DAE months (±1 SD)	5.2 (2.8)	18.9 (9.3)	16.3 (7.7)	18.1 (9.6)	15.6 (9.4)
	Range DAE months	1.0–8.0	7.0–38.0	6.0–25.0	5.0–30.0	1.0–38.0
	Mean DQ (±1 SD)	45 (27)	40 (17)	19 (10)	15 (9)	31 (20)
	Range DQ	6–79	16–69	6–31	3–25	3–79
Receptive language	Number	6	11	7	6	30
	Mean DAE months (±1 SD)	4.1 (2.7)	15.5 (9.3)	14.3 (10.7)	12.4 (12.4)	12.3 (10.0)
	Range DAE months	1.1–8.0	2.1–34.0	0.2–27.0	0.2–29.0	0.2–34.0
	Mean DQ (±1 SD)	35 (27)	31 (16)	16 (12)	10 (9)	24 (19)
	Range DQ	7–79	5–53	0–29	0–23	0–79
Expressive language	Number	6	11	7	7	31
	Mean DAE months (±1 SD)	5.2 (1.7)	14.8 (6.9)	11.9 (8.2)	11.2 (11.2)	11.5 (8.2)
	Range DAE months	2.2–7.0	3.2–30.0	1.2–21.0	1.0–34.0	1.0–34.0
	Mean DQ (±1 SD)	42 (9)	30 (12)	14 (10)	9 (9)	24 (16)
	Range DQ	34–59	8–44	1–26	1–29	1–59
Fine motor functioning	Number	6	11	7	6	30
	Mean DAE months (±1 SD)	6.5 (2.1)	19.3 (9.8)	17.7 (8.4)	18.5 (10.9)	16.2 (9.7)
	Range DAE months	4.0–9.0	7.0–35.0	8.0–27.0	6.0–31.0	4.0–35.0
	Mean DQ (±1 SD)	56 (24)	40 (18)	21 (11)	15 (9)	34 (22)
	Range DQ	26–89	16–71	8–33	3–25	3–89
Gross motor functioning	Number	5	11	7	6	29
	Mean DAE months (±1 SD)	9.0 (3.1)	19.3 (6.8)	18.7 (6.3)	17.8 (7.1)	17.1 (7.0)
	Range DAE months	4.1–12.0	9.0–33.0	9.0–27.0	6.00–26.0	4.1–33.0
	Mean DQ (±1 SD)	76 (24)	40 (9)	21 (8)	14 (7)	36 (24)
	Range DQ	59–119	20–54	10–29	4–22	4–119

*DAE* developmental age-equivalent, *DQ* developmental quotient

^a^Data of individual 31 were excluded from this table because WPPSI-III-NL results cannot be compared with the Bayley-III-NL. The cognitive DAE for this individual was 52 months

The developmental patterns of cognitive DAE and DQ were similar in the domains of language and motor development (Additional file 1: Fig. S1 and Table 2), but individual 31 was not included because results of the WPPSI-III-NL cannot be compared for these domains. Language development was more affected than other domains. Maximal DAE for receptive and expressive language was 34 months, with corresponding DQs of 53 and 29, respectively.

With respect to motor development, the ability to walk independently was acquired between 12 and 96 months of age (Table 1, *n* = 28, median 24 months, mean 29.4 ± 18.6 SD). Maximal DAEs for fine and gross motor domains were 35 and 33 months, with corresponding DQs of 55 to 51 (Table 2). However, individual 31 could not be compared on language and motor domains. She had a PIQ of 55 and a VIQ of 64, suggesting relatively higher performal and verbal functioning compared to the other children in this cohort but lower than the typical peers (PIQ and VIQ >85).

In general, children in our cohort performed poorest in the domain of language development and best in the domain of motor development (groups 1, 2, and 3) or cognition (group 4). There were no clinical differences between receptive (mean DAE 12.3, DQ 24) and expressive (mean DAE 11.5, DQ 24) language, nor between fine (mean DAE 16.2, DQ 34) and gross (mean DAE 17.1, DQ 36) motor function. At the individual level, however, 7/ 28 children (25 %) had a DAE that was at least 3 months higher for receptive language and 5/28 (18 %) had a DAE that was at least 3 months higher for expressive language, with maximum differences of 12 and 7 months, respectively (data not shown). Six out of 27 children (22 %) had a DAE that was at least 3 months higher for fine motor function and 9/27 (33 %) had a DAE that was at least 3 months higher for gross motor function, with maximum differences of 11 and 8 months, respectively.

### Developmental follow-up

Table 3 demonstrates the mean DG for the different age groups, i.e. the increase in developmental age in months per 6 months per domain. The DG is highly variable in each age group for all domains. The mean DG shows a similar pattern to the DQ: DG is highest in the first age group for the domains of cognition, receptive, and expressive language and fine motor functioning and decreases in subsequent age groups. The DG of gross motor functioning is highest in the second age group and then decreases.

**Table 3:** Bayley-III-NL developmental growth (DG) for each age group

Developmental domain		Age group 1 0–35.9 months	Age group 2 36–71.9 months	Age group 3 72–107.9 months	Age group 4 ≥108 months	All ages
Cognition^a^	Number	5	10	6	7	28
	DG in mo/6 mo, mean (±1 SD)	3.5 (3.2)	1.2 (2.0)	0.7 (1.0)	–0.9 (2.8)	1.0 (2.6)
	Minimum–maximum	0.6–7.0	−1.9–3.9	−0.4–2.4	−7.0–1.7	−7.0–7.0
Receptive language	Number	4	7	4	5	20
	DG in mo/6 mo, mean (±1 SD)	2.5 (1.5)	0.3 (2.5)	−0.4 (1.5)	0.1 (1.9)	0.6 (2.1)
	Minimum–maximum	0.5–3.8	−2.8–3.1	−2.0–1.2	−3.0–2.2	−3.0–3.8
Expressive language	Number	4	7	4	5	20
	DG in mo/6 mo, mean (±1 SD)	3.3 (1.5)	0.6 (2.4)	1.3 (0.9)	1.7 (2.5)	1.5 (2.1)
	Minimum–maximum	1.0–4.4	−4.4–3.1	0.0–2.0	0.0–6.0	−4.4–6.0
Fine motor functioning	Number	4	8	6	5	23
	DG in mo/6 mo, mean (±1 SD)	2.5 (2.5)	0.4 (3.8)	0.3 (1.2)	−0.5 (1.0)	0.5 (2.6)
	Minimum–maximum	0.0–5.7	−6.7–7.2	−1.2–2.1	−1.8–0.6	−6.7–7.2
Gross motor functioning	Number	5	8	5	4	22
	DG in mo/6 mo, mean (±1 SD)	1.4 (2.2)	2.0 (2.1)	−1.1 (2.4)	−1.0 (1.7)	0.7 (2.4)
	Minimum–maximum	−1.0–4.3	−1.0–5.1	−4.0–1.2	−2.0–1.5	−4.0–5.1

*DG* developmental growth, *mo* months

^a^Data of individual 31 were excluded from this table because WPPSI-III-NL results cannot be compared with the Bayley-III-NL. The cognitive DG for this individual was 0.9 months per 6 months

Five of 29 children (17 %) showed a cognitive DG of 0 and six (21 %) showed a negative cognitive DG ranging from −0.4 to −7.0 months per 6 months (data not shown). Children with no improvement in DG belong to age group 2 (*n* = 3), group 3 (*n* = 3) and group 4 (*n* = 5). Individual 31, who was assessed with the WPPSI-III-NL, showed a DG of 0.9. Only five children (17 %) showed a positive cognitive DG of more than 3 months (individuals 1, 5, 8, 15, and 19), and only one of them had a positive DG in all five domains (individual 1).

### Adaptive behaviour

In our study cohort of 33 children, there is a high proportion of children with deficiencies in adaptive behaviour, as indicated by the results of the VABS. DAEs increase most strongly between groups 1 and 2 (Table 4). Maximal DAEs for communicative skills, social skills, and daily skills (all for individual 31) were 51, 61, and 59 months, with a DQ of 43, 51, and 50, respectively. Similar to cognitive functioning, we found decreasing DQs with increasing age for all domains of adaptive behaviour. Twenty-nine children had a DQ < 85 in all domains (individual data not shown). Normal DQs were only observed for children from the youngest age group (individuals 1, 2, 3, and 6).

**Table 4:** VABS developmental age-equivalents (DAEs) and developmental quotients (DQs) for each age group

Developmental domain		Age group 1 0–35.9 months	Age group 2 36–71.9 months	Age group 3 72–107.9 months	Age group 4 ≥108 months	All ages
Communicative skills	Number	6	12	7	8	33
	Mean DAE months (±1 SD)	8.3 (3.0)	16.4 (7.5)	14.4 (6.2)	18.1 (15.6)	14.9 (9.6)
	Range DAE months	6–13	6–33	6–21	6–51	6–51
	Mean DQ (±1 SD)	70 (28)	34 (11)	16 (8)	14 (13)	32 (25)
	Range DQ	37–109	16–51	6–25	5–43	5–109
Social skills	Number	6	12	7	8	33
	Mean DAE months (±1 SD)	9.2 (4.5)	20.7 (8.9)	14.3 (9.4)	22.9 (19.6)	17.8 (12.6)
	Range DAE months	1–14	3–39	3–26	1–61	1–61
	Mean DQ (±1 SD)	72 (41)	44 (16)	16 (11)	17 (16)	37 (29)
	Range DQ	16–138	7–65	3–29	1–51	1–138
Daily skills	Number	6	12	7	8	33
	Mean DAE months (±1 SD)	11.7 (3.2)	20.8 (9.7)	21.3 (9.6)	25.3 (15.9)	20.3 (11.3)
	Range DAE months	10–18	10–39	10–35	14–59	10–59
	Mean DQ (±1 SD)	99 (34)	44 (15)	24 (13)	20 (13)	44 (34)
	Range DQ	59–156	24–64	11–49	9–50	9–156

Data on communicative, social skills and daily skills of the VABS

*DAE* developmental age-equivalent, *DQ* developmental quotient

### Deletion size and gender

**Fig. 2:**
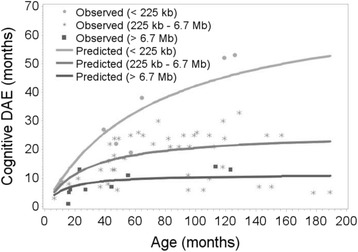
Relation between chronological age and cognitive developmental age-equivalents (DAE) for children (*n* = 32) with small deletions (group 1, *n* = 4), larger deletions (group 2, *n* = 23) and deletions including *PARVB* and *SULT4A1* genes (group 3, *n* = 5). There is a significant difference between groups 1 and 2 (*p* < 0.001) and between groups 2 and 3 (*p* = 0.044)

Figure 2 illustrates the relation between chronological age and DAE for children of different deletion groups. There is a significant difference between deletion groups (*p* < 0.001). The mean chronological ages at which children drop to a DQ of 50 (*θ*) was 73.1 months for group 1 (CI 56.6; 89.6), 26.0 months for group 2 (CI 19.9; 32.1) and 11.5 months for group 3 (CI 0; 24.3). There is also a significant difference between groups 1 and 2 (*p* < 0.001) and between groups 2 and 3 (*p* = 0.044). Thus, all deletion groups follow a different developmental path.

There was no clear difference between males and females with respect to mean DAE and DQ for the different age groups (Additional file 2: Table S1). The statistical analysis correspondingly showed a *p* value of 0.395 (data not shown).

## Discussion

Our study is a systematic and longitudinal evaluation of development in a relatively large cohort of 34 children with a 22q13.3 deletion (PMS), in which 33 children had a non-mosaic *SHANK3* deletion. For these 33 children, we reported results from different developmental domains. We are the first to report the influence of age at testing and, for 29 children, cognitive developmental growth by follow-up. In addition, we evaluated deficits in adaptive behaviour and deletion characteristics.

In our study group of 33 children, we found a global developmental delay in all children with maximal DAEs of 34 to 38 months (roughly 3–4.5 years), depending on the Bayley-III-NL developmental domain. However, one child (individual 31) showed maximal DAEs for all Bayley-III-NL domains and was additionally assessed with the WPPSI-III-NL. Although Bayley-III-NL and WPPSI-III-NL results cannot be entirely compared, both methods assess the level of cognitive functioning. Her DAE for cognition showed a higher level of developmental functioning with a cognitive DAE of 52 months (WPPSI-III-NL), increasing the range of cognitive functioning in our cohort. Other studies in children with 22q13.3 deletion syndrome reported similar cognitive age-equivalents of maximally 21 to 44 months [6, 18, 19], suggesting there is a maximal level of functioning that can be achieved in children with PMS. The severity of this intellectual disability prevents these children from passing through the stages of normal development. Before the age of 2 years, normal development in a typical child is largely sensorimotor-based and autonomic. Between the ages of 2 and 4 years, gross motor actions develop and word production supports concrete thinking. Acquired basic skills are combined and refined to more complex skills and higher order thinking. Between the ages of 4 and 7 years, fine motor skills become better, followed by discovery of abstraction of ideas and relations between actions [34]. In contrast, most children with PMS seem to acquire basic skills like walking, but subsequent extension and refinement of these skills is generally very limited, resulting in a developmental age-equivalent of 3 to 4.5 years in our study group.

Soorya et al. [19] reported that language was more severely affected than motor development in a group of 27 individuals with PMS. They additionally reported a better score for receptive than expressive language and a better score for gross than fine motor function, based on mean age-equivalents. When looking at all ages in our cohort, children did seem to function slightly better in receptive rather than expressive language, but overall, we saw a slightly better fine than gross motor function. Looking in more detail at individual levels, however, these findings were not consistent and very variable because the extent of these differences ranges from limited to more pronounced and because it can be directed to either of the two subdomains of language and motor function domains.

We also report that the intellectual disability is less striking in younger children with 22q13.3 deletion syndrome than in older children, as relative developmental functioning (DQ) decreases with increasing age. This relative decrease in developmental functioning is known as ‘growing into deficit’ [35] and may, in part, be explained by the fact that developmental requirements are lower in the first few years of life when movement is only in a one- or two-dimensional direction and language mainly passive. In this stage, developmental differences compared to normal peers are smaller and less apparent. This is an important observation that indicates that by combining DQs or IQs of older and younger children, the severity of intellectual disability of individuals with PMS may be underestimated (Table 2, last column). A comparable decrease in relative functioning with increasing age is known but not as striking, for more common intellectual disability disorders like Down syndrome (trisomy 21) [36] and 22q11.2 deletion syndrome [37]. In Down syndrome, mean IQ decreases from approximately 50 to 35 (a 30 % decline) between 5 and 10 years of age and in 22q11.2 deletion syndrome from 80 to 70 (a 12 % decline). In our PMS cohort, the mean decrease in DQ of 39 in age group 2 and 19 in and group 4 represents a decline of 50 %. This difference might be explained by a higher level of social/adaptive functioning in Down syndrome and of cognitive functioning in 22q11.2 deletion syndrome. Individuals with PMS have both cognitive and adaptive behaviour deficits, as demonstrated by our results. The two children with better adaptive behaviour (individuals 2 and 3) clearly had higher DQs for cognition (79 and 73 vs. 6–38), receptive language (79 and 55 vs. 7–25), and fine motor function (89 and 73 vs. 23–51) than the other children of their age group, but they also had smaller deletions. Interestingly, neither of them had a high DQ for expressive language (59 and 38 vs. 27–53). The remaining questions in our population are whether deficits in cognitive functioning lead to limited adaptive behaviour and whether problems with adaptive behaviour further hamper cognitive development [38], especially in the developmental phase where progress depends on abstract and relational thinking. Both cognitive deficits and adaptive behaviour have an effect on language development, so, either way, it is likely that children with this combination have more difficulties reaching a certain level of developmental functioning.

Our most disconcerting observation, however, is that 11 of our 29 children show no improvement of cognitive developmental functioning. This finding might indicate stagnation or even regression in these children, which support the reports of loss of skills we often receive from parents. A possible explanation for this absence of cognitive improvement in PMS might be the co-occurrence of acute events. It has been reported that the loss of skills in PMS is associated with infection, epilepsy, or malignant neuroleptic syndrome [4]. Although we do not have any data that suggests this was the case in our cohort, we cannot fully exclude this possibility. Systematic developmental studies with longer follow-up and larger sample sizes in children and adults should be performed to investigate whether stagnation and regression are indeed part of the developmental phenotype in individuals with Phelan-McDermid syndrome. These studies might also identify whether stagnation of development is intrinsic to the *SHANK3* deletion or if it can be attributed to problems in adaptive behaviour, acute events or other factors.

Our results also show that the extent of developmental deficiencies in children with 22q13.3 deletion syndrome is highly variable within age groups, suggesting a contribution of factors other than *SHANK3* haploinsufficiency to the developmental phenotype. Based on statistical approaches, Sarasua et al. 2013 [39] suggested a relation between deletion size and level of phenotype severity. We show that children with the smallest deletions (those only including the *SHANK3*, *ACR*, and *RABL2B* genes) do function at a relatively higher level (Fig. 1 and Additional file 1: Fig. S1). Thus there might be an additive negative effect on development by one or more genes located outside this smallest region, although it is not yet known which additional genes contribute to the phenotype. The child with a mosaic *SHANK3* deletion and the five children with a ring chromosome also seem to function at a higher level within their age group (Fig. 1 and Additional file 1: Fig. S1). It is not surprising that a mosaic deletion results in an ameliorated phenotype because not all cells contain the deletion, but even this individual has significant deficits in adaptive behaviour. Why the children with a ring chromosome seem to function better is not clear. It may partly be explained by the limited deletion size of the ring chromosomes, which are all between 2.3 and 3.4 Mb and thus represent a smaller number of missing genes. Disciglio et al. 2014 [16] discussed the effect of haploinsufficiency of *SULT4A1* and *PARVB* genes on neurological features of individuals with an interstitial 22q13.3 deletion that did not include *SHANK3*. In our group, these genes are not included in the deletions of the children with a ring chromosome, but they are in the deletions of individuals from our deletion group 3, who all have a terminal deletion size ≥7.3 Mb. These group 3 children have lower DAEs (Fig. 2) and cognitive DQs (data not shown). However, at the individual level, the larger deletions including *SULT4A1* and *PARVB* genes do not predict the level of cognitive functioning of children because DAEs and DQs of children from deletion group 2 overlap with those from deletion group 3. In addition, looking at individual results within the age groups, the additive effect of *SULT4A1* and *PARVB* is inconsistent. In age groups 1 and 2, individuals with a *SULT4A1* and *PARVB* deletion had a lower DQ than individuals without deletion of these genes (age group 1: individual 4 (DQ 31) and 5 (DQ 35) vs. individual 1 (DQ 48); age group 2: individual 14 (DQ 16) vs. individuals 12, 13, and 15 (DQs between 38 and 51)), but in age group 4, the individual with a deletion of these genes has a higher DQ (individual 29 (DQ 12) vs. individuals 32 and 34 (7 and 5)). One explanation might be a progressive effect of *SHANK3* haploinsufficiency on intellectual functioning at older age. This could lead to a less additive effect of the deletion of other genes and thus a decrease of the deletion size effect at older age. However, our findings and explorative statistical analysis concern a relative small sample size with small subgroups and an additional effect of *SULT4A1* and *PARVB* cannot fully be excluded or supported by our study. Despite the significant results, we consider these results not as a confirmation but as stimulation for researchers to consider the effect of deletion size and more specifically gene content on developmental functioning. A larger study sample is needed to prove this hypothesis.

In addition to the effect of age and deletion size, we also compared mean cognitive DAEs and DQs between males and females (Additional file 2: Table S1) and performed an explorative statistical analysis. Although deletion size was not included and subgroups are too small to draw conclusions, we did not see a consistent difference between males and females. Analysis in larger samples could also include deletion size and more reliably explore any differences that depend on gender.

Several limitations need to be considered in interpreting our results. The number of participants in each age group is small, and we could not obtain developmental data on every domain from each child. There are several reasons for these missing data. One is that the assessment of children depends on their cooperation. There is a high prevalence of behavioural problems in the autism spectrum in individuals with PMS, such as a reduced interest in social interactions and a short attention span [19]. This makes it more difficult to obtain and keep their attention during testing. In addition, we used a screener version of the VABS. A more extensive test or a second test to assess adaptive behaviour could complement and support our findings. However, the section of the Bayley-III-NL questionnaire that assesses adaptive behaviour is less specific and very time-consuming. In consequence, we decided to only use the screener version because this would be sufficient to indicate the level of developmental functioning of adaptive behaviour and limit the burden of the assessments. Another issue is that children with PMS tend to lose acquired skills, at least temporarily, in the course of an illness or other disturbance of their wellbeing, sometimes without a clear explanation [19]. This illustrates that one assessment of development in these children does not always reliably represent cognitive functioning and it is therefore recommended that several assessments be performed longitudinally.

## Conclusions

Our study shows that cognitive, motor and especially language development are significantly impaired in all children with 22q13.3 deletion syndrome including *SHANK3* as compared to children with more common chromosomal disorders. These deficiencies are less striking in younger than older children, a phenomenon known as ‘growing into deficit’. Very small 22q13.3 deletions, i.e. those including only the *SHANK3*, *ACR*, and *RABL2B* genes, have a more favourable developmental phenotype. In addition, deficits in adaptive behaviour further hamper cognitive development. No improvement of developmental functioning is a common finding in this population, and this is important to take into account when evaluating development and treatment effects. In particular, cognitive and behavioural characteristics should be evaluated and followed in each child with PMS to adapt supportive and therapeutic strategies to meet individual needs. Further research evaluating the relation between deletion characteristics and the developmental phenotype is warranted to improve counselling of parents. Our results contribute to an improved insight in the developmental phenotype of children with PMS and hopefully support health care professionals in counselling and therapeutic strategies.

## Additional files

Additional file 1: Figure S1. Developmental age-equivalents (DAE) **A**, **C**, **E**, **G** and developmental quotients (DQs) **B**, **D**, **F**, **H** of children with 22q13.3 deletions. Data based on Bayley-III-NL (*n* = 33) results for receptive language, expressive language, fine motor function, and gross motor function. The dashed line represents the normal relation between DAE and age in typically developing children. Terminal deletion = terminal deletion not caused by ring chromosome formation. Mosaic deletion (individual 7). Additional CNV = additional copy number variation (individuals 5, 16, 28). S = small deletion including only *SHANK3*, *ACR*, and *RABL2B* (individuals 10, 17, 18). (EPS 2.05 mb) Additional file 2: Table S1. Bayley-III-NL developmental age-equivalents (DAEs) and developmental quotients (DQs) for cognition separated by gender. (XLS 18.0 kb)

## Abbreviations

Bayley-III-NL: Bayley Scales of Infant and Toddler Development, third edition, Dutch version
CA: chronological age
CNV: copy number variation
DAE: developmental age-equivalent
DG: developmental growth
DQ: developmental quotient
FSIQ: full scale intelligence quotient
PIQ: performal intelligence quotient
PMS: Phelan-McDermid syndrome
VABS: Vineland Adaptive Behavior Scales
VIQ: verbal intelligence quotient
WPPSI-III-NL: Wechsler Preschool and Primary Scale of Intelligence, third edition, Dutch version
